# Spectroscopic Understanding of SnO_2_ and WO_3_ Metal Oxide Surfaces with Advanced Synchrotron Based; XPS-UPS and Near Ambient Pressure (NAP) XPS Surface Sensitive Techniques for Gas Sensor Applications under Operational Conditions

**DOI:** 10.3390/s19214737

**Published:** 2019-10-31

**Authors:** Engin Ciftyürek, Břetislav Šmíd, Zheshen Li, Vladimír Matolín, Klaus Schierbaum

**Affiliations:** 1 Department of Materials Science, Institute of Experimental Physics and Condensed Matter, Heinrich Heine University Düsseldorf, 40225 Düsseldorf, Germany; 2 Department of Surface and Plasma Science, Faculty of Mathematics and Physics, Charles University, V Holešovičkách 2, 18000 Prague 8, Czech Republic; 3 Department of Physics and Astronomy—Centre for Storage Ring Facilities (ISA), Aarhus University, 8000 Aarhus, Denmark

**Keywords:** metal oxides, gas sensors, spectroscopy, characterization techniques, synchrotron, XPS, NAP-XPS

## Abstract

The most promising and utilized chemical sensing materials, WO_3_ and SnO_2_ were characterized by means advanced synchrotron based XPS, UPS, NAP-XPS techniques. The complementary electrical resistance and sensor testing experiments were also completed. A comparison and evaluation of some of the prominent and newly employed spectroscopic characterization techniques for chemical sensors were provided. The chemical nature and oxidation state of the WO_3_ and SnO_2_ thin films were explored at different depths from imminent surface to a maximum of 1.5 nm depth from the surface with non-destructive depth profiling. The adsorption and amount of chemisorbed oxygen species were precisely analyzed and quantified as a function of temperature between 25–400 °C under realistic operating conditions for chemical sensors employing 1–5 mbar pressures of oxygen (O_2_) and carbon monoxide (CO). The effect of realistic CO and O_2_ gas pressures on adsorbed water (H_2_O), OH^−^ groups and chemisorbed oxygen species ($O_{2\left(ads\right)}^{-},\;O_{\left(ads\right),\;}^{-}O_{2\left(ads\right)}^{2-}$) and chemical stability of metal oxide surfaces were evaluated and quantified.

## 1. Introduction

The sheer size of the global gas sensor market valued at $2.05 billion last year and expected to increase up to $3.7 billion by 2025. Gas sensor technology has found sound application in a variety of fields ranging from safety related applications to process monitoring, online decision mechanism requiring artificial intelligence, energy applications, environmental safety, smart city regulations, in-situ monitoring of solid oxide fuel cells, gasifiers, and the food industry [1,2,3,4]. The commercially available sensor designs are composed of electrochemical, semiconducting oxide type, catalytic, infrared, photo ion detector type detection systems. Semiconducting metal oxides (SMOs) in the form of thin/thick films cover up to 40% of the material sets utilized in the sensor market. The level of pressure on engineers and scientists has been continuously increasing to satisfy the demands of the market since early the 2000s for especially SMOs type chemicals sensors due to their simplistic and robust nature, their convenience for miniaturization and in-situ applicability [5]. However, that macroscopically simple looking working principle brings complicated multi-lateral chemical and physical processes together with a high level of variety in operation conditions such as target gas pressure/composition, operation temperature, level of humidity, interfering gases, oxygen content and wide range of material sets utilized as sensing material. 

The quantitative model for semiconducting metal oxide gas (SMO) sensing mechanism achieved in the last 40 year with contributions from engineers, scientists from broad range of disciplines and industrial partners consolidated the knowledge derived from ceramic and thin/thick film processing, semiconductor physics, elementary chemical reactions and surface science provided basic description of gas sensing principle despite having shortcomings. The reduction-oxidation (redox) reactions governing the functionality of the SMOs based chemical sensors happen to be on the first several nanometers (nm) of the sensing material. It is well known that a significant contribution to the quantitative model is attained from ultrahigh vacuum (UHV) based techniques. The data used for developing that quantitative model has been collected from functional oxide ceramics in the form of thin/thick films utilized for chemical sensors, solid oxide fuel cells (SOFCs), superconductors, batteries, and catalysis research. However, it should be noted that the data collected under the measurement conditions is unrealistic compare to the real operation conditions, as many surface technique suffers from pressure and temperature limitations for being implemented as an in-situ analysis technique for chemical sensor testing. 

There has been great progress in the analytic and spectroscopic techniques applied for analysis of chemical sensing with metal oxides since then the first invention of the gas sensor dating back the 1960s. There are many methods out in the literature frequently have been employed for analysis of material property, microstructure, chemical purity, phase, structure and sensing mechanism in semiconducting metal oxide (SMOs) based chemical sensors. The most frequently used techniques are XRD, TEM, EDS, SEM, FT-IR, XPS, UPS, UV-Vis, Raman, EPR, NMR, PL, LEED, Mössbauer, XAS and XES. A very few of those techniques satisfy the requirements for the in-situ analysis together with surface sensitivity required for the semiconducting metal oxide (SMO) chemical sensor surface analysis. Many of those techniques are qualified as surface averaging techniques, with poor lateral resolution in the range of µm to cm with mainly bulk analysis capability. Apart from XPS and UPS, all methods listed above are designated as bulk characterization approaches from the perspective of a chemical sensor designer [6,7,8]. The FT-IR and Raman are mainly used for molecular analysis, defect structure, doping and purity of oxides and metals by analyzing spectral features produced by nearest neighbor interactions and vibrational modes. Given the fact that, data acquisition depth in both FTIR and Raman are in the range of micrometers (µm), it would not be possible to collect information from the sensor surface regarding the oxygen species such as lattice and surface adsorbed oxygen ions and chemical status of the sensing material. The most common two UV-Vis based techniques are Photoluminescence (PL) and Modulation Spectroscopy (MS). Both techniques can probe from 50 nm to several micrometers depth from a surface. UV-Vis is mainly used on semiconductors, as the peak width, intensity and positions leading to the band gap, concentration and identification of impurities with very low level of detection limit with user friendly instrumentation while lacking of chemical state analysis, high temperature compatibility and surface sensibility, in best case 1 µm minimum depth data acquisitions were achievable and in very special cases 100 nm depth analysis was achieved.

The tunable excitations energy nature of synchrotron based X-rays allows other spectroscopic techniques that complementary to XPS-UPS, referred to generically as X-ray absorption spectroscopy (XAS). There are multiple techniques belonging to XAS family. X-ray absorption fine structure (XAFS) is capable of examining the chemical bond lengths, molecular orientation and coordination of the chemisorbed oxygen ions to analyze oxygen ion network on the surface. Contrary to the XPS, the XAFS may show distinct peak formation separated by several eV belonging to interstitials oxygen ions in the bulk, on which XPS is not capable of, yet XAFS is generally not considered quantitative technique based on the fact that extracted electrons are integrally collected. The XAFS spectrum has two distinct parts, the X-ray absorption near-edge structure (XANES), covering the energy range up to 50 eV above the atomic absorption edge, and the extended X-ray absorption fine structure (EXAFS) region above this photon energy. Structural information of molecular arrangements, coordination and adsorbed species are the main targets for these techniques [9]. These techniques have the drawback of having a limited quantitative analysis of elemental/chemical composition. EXAFS is not considered to be surface sensitive technique, also does not have oxidation state analysis capability and also brings high photon damage probability. Surface-extended X-ray absorption fine structure (SEXAFS) is just for surface sensitive structural information by analyzing Auger electrons as a function of the incident photon energy. SEXAFS and XANES are both considered to be surface sensitive techniques, on the other hand they both do not provide quantitative results of the chemical status of the constituent elements of the compound or the chemisorbed species. In comparison to the XAS family, the unique advantage of the XPS-UPS technique is the high precision in the quantification through well-established peak deconvolution analysis for distinguishing different chemical states of elements. On top of this, it is highly possible to conduct depth monitoring of the chemical state of constituent element through the structure by changing photon energy and/or electron take off angles. The absolute work function values can be solely measured via UPS, while a comparison type of approach is Kelvin probe setup, as the latter provide changes of work function. UPS is the most powerful and versatile technique to study the electronic structure, band bending, defect states, shift in the valence band and determination of work function (Φ) changes. Mössbauer spectroscopy is another way to quantify the oxidation state if the material possesses the Mössbauer effect actively, however it requires drastic working environments such as gamma source radiation, gyroscopic temperatures, and can be practically considered as an extreme difficulty to realize for a chemical sensor designer.

The majority of the literature dealt partially or fully with the chemisorption of the oxygen on metal surfaces as a molecular (O_2_) absorption on catalytically active precious metals such as silver (Ag), palladium (Pd), platinum (Pt) under ultra-high vacuum (UHV) using low levels of gas dosing pressures like 10^−5^–10^−8^ mbar. Those studies also combined temperature programmed reduction (TPR) studies with surface crystal structural analysis methods such as LEED with UHV based XPS-UPS for understanding the chemisorbed species [10,11,12]. Interaction of the oxygen species with H_2_O, CO, H_2_ and other organic chemicals under UHV conditions was also investigated. However, because of technical limitations, polycrystalline thick/thin films and their interaction with adsorbed oxygen species, lattice oxygen ions and their interaction with the gas species in question at higher temperatures under realistic working conditions/pressures could not be realized. The detailed explanation on the subject of the formation and structure of oxygen adsorbates on the clean single crystal metals have been well detailed in the literature regarding surface coverage, concentration, rate limiting factors and diffusion related phenomenon [11,12,13].

X-ray photoelectron spectroscopy (XPS) is one of the most frequently used surface sensitive spectroscopic techniques capable of elemental and chemical analysis of the very first few atomic layers (up to 10 nm) of a solid surface. Despite the frequent use of XPS as a spectroscopic tool for the characterization of materials, owing to technical and physical limitations stemming from short electron mean free path distances in the extremely dense gas environment, the utilization of XPS for chemical sensor applications limited to unrealistic UHV conditions rather than real chemical sensor operational environment. Most of the electron transport demanding surface science approaches necessitates the measurement chamber operation pressure to be in the range of 10^−8^–10^−10^ mbar, while spectroscopic techniques involving in photon transfer and/or reflectance-absorbance do not require UHV conditions. The information gathered from photon transfer techniques such as UV-Vis, FTIR, Raman, EPR, XAS in general do not represent the chemical status directly more importantly, they do not provide surface sensitive information required for understanding chemisorbed oxygen species. Electron paramagnetic resonance (EPR) is capable of detecting the paramagnetic oxygen ions such as OH^−^, O^−^, O^2−^ on metal oxides, however this technique requires temperatures as low as −200 °C, multiple treatment of the sample surface in addition to the fact that information collected is representative of bulk rather than a surface analysis. Essentially near-ambient pressure X-ray photoelectron spectroscopy (NAP-XPS) provides an option for in-situ analysis under realistic operation conditions relevant to semiconducting metal oxide (SMO) based chemical gas sensors in combination with high surface sensitivity and quantitative analysis capability. The first time XPS investigations of different adsorbed oxygen species and lattice oxygen ions within 1 nm depth from a chemical sensor surface under realistic operational conditions was achived by utilizing NAP-XPS recently realized by Ciftyürek et al. [14,15,16,17]. In that work adsorption of oxygen species ($O_{2\left(ads\right)}^{-},\;O_{\left(ads\right),\;}^{-}O_{2\left(ads\right)}^{2-}$), onto metal oxide surfaces were analyzed as a function of temperature and partial pressure of oxygen and carbon monoxide (CO) [14].

Some of the surface sensitive techniques mentioned in the preceding paragraphs have been applied for chemical sensors analysis, however information gathered did not provide an understanding related to surface adsorbed oxygen species and oxygen sublattice as those oxygen species provide ultimate functionality to a chemical sensor. The importance of chemisorbed oxygen species emerges from the very critical part they perform in the sequence of chemical sensing functionality of metal oxide based sensors [18]. The chemical sensing mechanism is explained on the basis of the consumption of chemisorbed oxygen species by target gas molecules (H_2_, CO, H_2_S, SO_2_, etc.) on the chemical sensor surface. It is very difficult to characterize the chemisorbed oxygen species ($O_{2\left(ads\right)}^{-},\;O_{\left(ads\right),\;}^{-}O_{2\left(ads\right)}^{2-}$) under working conditions due to very low concentration of them located on the outermost part of the chemical sensor surface blended with oxygen vacancies and lattice oxygen ions. The existence of oxygen vacancies not only regulates the electronic structure of metal oxides, but also provides more active sites for adsorption of oxygen molecules. The forms of adsorbed ionic oxygen are superoxide ($O_{2\left(ads\right)}^{-})$, monoatomic ($O_{\left(ads\right)}^{-})$ ion and double charged superoxide ($O_{2\left(ads\right)}^{-})$ ions. A number of experimental observations indicate that adsorbed oxygen species on the metal oxide surface increases their electron concentration if the temperature and surface properties of the metal oxide facilitates [19]. The process of incorporation of chemisorbed oxygen into the lattice requires higher temperature and it is out of the scope of this paper.

The current work aims to study functional chemical sensor grade thin film metal oxides, under realistic gas sensor working conditions. Two of the most commonly employed sensing materials SnO_2_ and WO_3_ were used in this work with advanced state of the art surface sensitive techniques. Besides in-situ understanding/quantification of oxygen adsorption and consumption, it is also important to understand the electronic and chemical properties on the surface. The conditions of the measurements, including temperature, gas composition, and oxygen levels and target gas pressures kept as close as to the actuals application conditions. Additionally, current work aimed to show applicability of some state of the art characterization methods and some practical notes concerning the several pitfalls and addressing some misconceptions commonly found in the literature regarding the analysis/quantification of the XPS measurements for the characterization of metal oxide chemical sensors surfaces. In order to realize those aims; firstly, Low-dose PM4 equipped with ARTOF analyzer was utilized in order to precise measurements of oxygen (O) 1s and tungsten (W) 4f main photoelectron lines in order to determine the corresponding photoelectron binding energies for radiation damage sensitive chemisorbed oxygen species, lattice oxygen ions, and oxidation states analysis of tungsten species. Low electron energy microscope (LEEM) was utilized to understand morphology and local stoichiometry on the thin film surfaces. Secondly, synchrotron based angle resolved XPS-UPS was employed in order to monitor oxygen species concentrations and the oxidation state of the tungsten (W) through the depth of the chemical sensor thin film. Varying X-ray excitation photon energies were used in order to probe the thin film through depth starting from the imminent surface down to 10 nm depth with a non-destructive/non-invasive manner. 

It is of significant importance to understand the surface composition of the metal oxide sensing material; due to the fact reduction-oxidation (redox) reactions governing the chemical sensing occur on the surface. NAP-XPS is a unique technique capable of conducting this type of analysis under realistic conditions. However, in many cases the evaluation and the interpretation of the experimental data measured under ultra-high vacuum (UHV) do not reflect the real situation (sensor working conditions). It is even more crucial to quantify the surface chemistry of the chemical sensor surface during operation since the axiomatic sensing explanations are based on such data. In order to comprehend a realistic interpretation of photoelectron spectroscopic (XPS-UPS) data, certain pitfalls must be avoided or at least potential avoidance and/or remediation methods should have been as minimally included in the intellectual data interpretation level by chemical sensor designers. NAP-XPS was utilized with the experience and knowledge extracted from preceding steps in order to quantify and understand the target gas and semiconducting metal oxide (SMO) sensor surface during an interaction under realistic working conditions for chemical sensors. Specific attention was directed to the interaction of chemisorbed and lattice oxygen ions with carbon monoxide (CO). It has never reported the surface chemical status of the adsorbed species and metal at the time of exposure. The changes in chemical status of the surface may be responsible for the switch behaviour in sensing response type from n-type to p-type in metal oxides reported in the literature in multiple cases. In those cases, it was reported that upon exposure to reducing gases of H_2_S and SO_2_, the p-type response was observed instead of the typical expected n-type response [3,20].

## 2. Materials and Methods

Plasma enhanced atomic layer deposition (PEALD) of SnO_2_ was performed in a custom-built stainless-steel reactor. The reactor consists of a square chamber with a top flow geometry for precursor/gas delivery equipped with a grounded substrate holder located between the antennas. Direct electron cyclotron wave resonance O_2_ plasma was generated by 13.56 MHz radio frequency generator with an active magnetic flux density of 2.8 mT using a matching network within the pressure range of 10^−2^–10^−3^ mbar. The plasma power was adjusted to 200 W, whereas O_2_ (oxygen) (Air Liquide, 99.995%) and Ar (argon) (Air Liquide, 99.995%) gas flows were adjusted to 15 sccm for all depositions. P-type Si (100) wafers served as substrates for depositions. The precursor was heated up to 140 °C, as plasma pulse lengths were kept at 500 ms. The WO_3_ thin films were grown at 600 °C and 700 °C substrate temperatures via metalorganic chemical vapour deposition (MOCVD) in a custom-built horizontal cold-wall CVD reactor. Silicon wafer and platinum (Pt) interdigitated electrodes (IDE) were used as substrates. The WO_3_ and SnO_2_ samples were characterized by vdP, XPS-UPS, LEEM, NAP-XPS. An in house designed 4-point probe technique was operated for high accuracy van der Pauw (vdP) electrical resistivity measurements in between 25–300 °C range. During vdP measurements samples were located in MACOR ceramic sample holder (Corning Inc., NY, USA) and heated from backside with computer controlled heating cycles. The temperature was measured via Pt-100 element. The 5 × 5 mm square geometry samples were used and the contacts were tungsten carbide (WC) probes located on the corners of the sample surfaces within a distance no more than 500 µm far away from the corners. The geometry of sample and probes heads was designed to minimize the error in measurements down to 0.1%. The data collection was completed with high resolution National Instruments (NI, TX, USA) PXIe-1071 digital mustimeter (DMM). The XPS-UPS investigations were performed using the Material Science Beamline (MATLINE) located at Aarhus University synchrotron facility, ASTRID2. SMART and PM4 instruments of BESSY II synchrotron facility were also employed. NAP-XPS measurements were conducted at Charles University, Prague. During the NAP-XPS measurements, heating was accomplished by electron beam heating from the back of the samples and the temperature measurement was completed with the thermocouple attached to a metal sample holder. During heating, O_2_ flow was maintained in the chamber and carbon monoxide (CO) was introduced as the oxygen flow terminated. The gas testing experiments were conducted at 100 °C substrate with different exposure times and concentrations of target gas. The NO_2_ testing was realized with NO_2_/dry air mixture and the N_2_ (nitrogen) was utilized between the pulses. The sensor response is defined as presented in Equation (1), in which Ia is the current measured without gas exposure, and Ig is the current measured with target gas. The response time is defined as the time taken by a chemical sensor to achieve 90% of its total resistance change as an adjustment in the concentration of the test gas.

(1) $$Sensor\;Response\equiv \left(S\right)=\left(\frac{Ig-Ia}{Ia}\right)\times 100=\left(\frac{\Delta I}{Ia}\right)\times 100$$

## 3. Results

### 3.1. Syncrothon Based XPS Analysis of WO_3_ for Precise Positioning of Chemisorbed Oxygen Species and Some Remarks on Oxidation State Analysis of WO_3_

Tungsten has abundant forms of stabile oxides and suboxides including but not limited to WO, WO_2_, W_2_O_3_, W_4_O_3_, W_18_O_49_ and WO_3_, as oxidation states ranging from W^2+^ to W^6+^ without taking integer numbers necessarily. Owing to this, oxides of tungsten preserve catalytic activity toward different gases. WO_3_ is an insulator in a stoichiometric form, however, the non-stoichiometric forms (WO_3−x_) shows an n-type semi-conduction. It is one of the most utilized semi-conducting metal oxide materials as a chemical sensor. There are numerous reports concerning the stoichiometry, porosity, density, thickness, roughness, electrical properties of WO_3_ thin/thick films produced by multi-disciplines [2,4,21,22,23,24,25]. There are very few reports on the precise analysis of the WO_3_ oxidation state as function of depth from the thin film surface. In this work, surface chemistry and homogeneity supported by spatially and laterally resolved insight into the chemical, electronic and electrical properties of the WO_3_ films were evaluated by means of synchrotron based XPS-UPS, LEEM and vdP.

#### 3.1.1. LEEM Analysis

**Figure 1:**
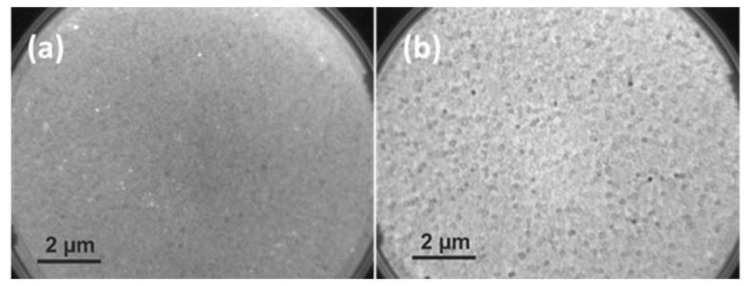
LEEM images of 600 °C (**a**) and 700 °C (**b**) deposited samples, the images were collected at field of view (FoV) of 12.4 µm with 5 eV kinetic energy.

LEEM analyzes surfaces by monitoring elastically backscattered electrons. LEEM was utilized to image the surface morphology, resolve thickness variations with atomic precision and domain structure, to characterize the local physical and chemical structures. At low kinetic electron energy, image contrast in LEEM micrographs can be determined by work function (Φ) differences, topography, different crystal facets polycrystalline materials. Surface topography, grain boundaries, porosity and stoichiometry can affect the work function as well thus contribute the image contrast in LEEM. LEEM correlates the surface topography to the difference in electronic properties and that leads to different visual construction of the surface topography in comparison to SEM. The bright-field LEEM images of WO_3_ at deposited at 600 °C and 700 °C are shown in Figure 1a,b, respectively. As the figure reveals the deposition at 600 °C forms a continuous thin film with homogeneous grain structure in size through the surface, whereas for the 700 °C deposited sample average grain size is larger in size with an obvious higher surface roughness, additionally abnormal grain growth occurred which was driven by higher deposition temperature leading to heterogeneous grain size and distribution. The variation in the work function (Φ) from grain to grain is higher in 700 °C compared to the 600 °C deposited sample due to different film growth time and temperature. The higher differential variation in the work function (Φ) especially in the 700 °C deposited sample attributed to the local distortions in intergrain contacts, grain growth and protruding features of the surface resembling a cauliflower type of growth in physical vapor deposited thin films.

#### 3.1.2. Low-Dose PM4 Synchrotron Analysis of WO_3_

Inorganic materials, particularly salts and oxides, such as those used in this work WO_3_ and SnO_2_, are sensitive to photon induced damage, such as reduction of oxide phase to the metallic state after prolonged high intensity X-ray exposure. It is expected that high intensity X-ray beam would have more powerful effect on undesired removal of the weakly bonded surface chemisorbed oxygen ions from the surface. On the other hand, it is required high enough X-ray photon intensity by considering the low concentration of the defect sites and adsorbed oxygen species in order to distinguish oxygen chemisorbed species from other oxygen ion containing functional surface groups such as OH^−^/H_2_O and lattice oxygen with XPS. PM4 station located at BESSY II equipped with a novel angle-resolved time-of-flight (ArTOF) spectrometer. Distinctive features of it are the extreme high transmission and the possibility to collect electrons over a very broad angular range in parallel. Thanks to the high transmission of the ArTOF, roughly 1000 times faster acquisition times and equally reduced dose rates could be achieved with respect to the more conventional hemispherical analyzers, thus making possible the XPS investigations of materials that usually suffer from radiation damage such can be encountered in WO_3_ and SnO_2_ cases. The spectral resolution achievable in synchrotron sources (0.1–0.2 eV) is much better than available in laboratory scale X-ray sources (1.0–2.0 eV). Due to 1–2 eV difference in the binding energy of the H_2_O/OH^−^, chemisorbed and lattice oxygen ions, it is important to have very high resolution such can be found in the synchrotron sources. By taking these considerations into account, PM4 was used to determine the core electron binding energies and full with half maximum values (FWHM) of O 1s and W 4f main photoelectron lines. 

**Figure 2:**
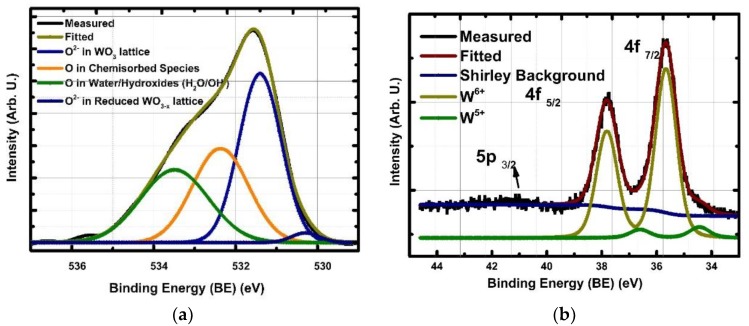

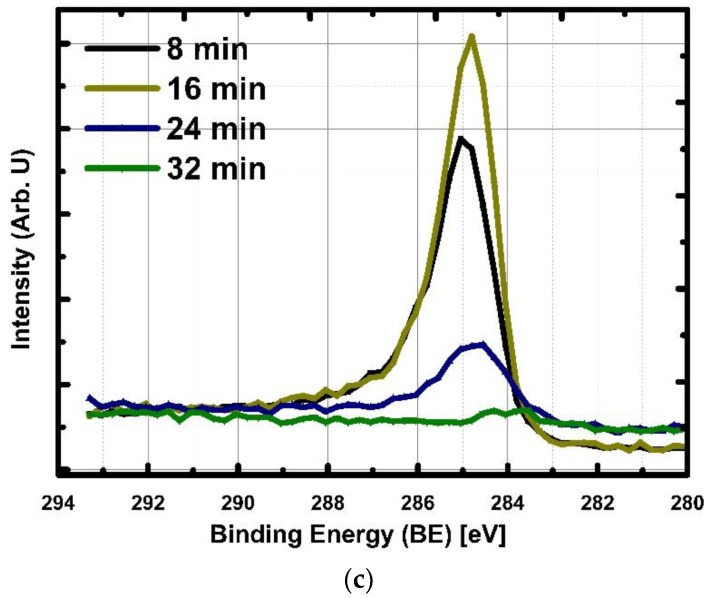
XPS main photoelectron lines of (**a**) oxygen (O) 1s and (**b**) tungsten (W) 4f (**c**) carbon (C) 1s measured at 25 °C under UHV conditions with 650 and 120 X-ray photons for baseline establishment of fitting parameters for further detailed quantification analysis. C 1s measurements was conducted under 10^−6^ mbar O_2_ partial pressure at 200 °C to clean the surface from carbon groups without damaging the surface stoichiometry.

Figure 2a–c shows the oxygen (O) 1s, tungsten (W) 4f and carbon (C) 1s and main photoelectron lines, respectively. Figure 2a shows the deconvolution analysis of the O 1s. It can be seen from the image that, the O 1s position was deconvoluted into four individual peaks, those are lattice (WO_3_) and reduced lattice (WO_3−x_), chemisorbed species and water/hydroxides. Table 1 provides the corresponding binding energies, peak properties and the concentration of each phase in details. The outermost layer of thin films typically contains carbon including oxygen containing functional groups, such as C–O and O–C=O, it is necessary to correct the O 1s photoemission for the contributions from these two species in order to increase precision in the distinction between oxygen species on the surface relevant to chemical sensing operations. As can be seen in the C 1s spectrum given in Figure 2c, the amount of carbon diminished to zero level after surface was treated with optimized conditions for WO_3_ at 200 °C with 10^−6^ mbar oxygen background by successive applications of 8 min intervals thus the total application time sums up to 32 min. During this cleaning procedure, W 4f line was monitored in order to detect any compositional change occurred or not. Based on this, it was determined that, there was not chance in the W 4f line, so it was concluded that, the cleaning process did not alter the stoichiometric status on the surface as well as did not create artificial features. Based on this, contribution from C–O bonds was not included further into the analysis. Figure 2c shows the W 4f analysis, as can be seen from the image, W^6+^ was detected as a majority phase in addition to the W^5+^ minority phase.

Table 1 summarizes all the results obtained for O 1s and W 4f measurements from the WO_3_ film. The binding energies for O 1s and W 4f were calculated by subtracting the photon energy from the measured kinetic energies. By adjusting the spectrometer work function, the binding energy position of the fermi edge of a gold (Au) foil was corrected to 0 eV. The photon energies used in the experiments were 650 eV and 120 eV for O 1s and W 4f, respectively. The slit size of the incoming photons was 100 and 200 µm, natural broadening of X-ray photons was 0.06 eV. The peak fitting was accomplished using the KolXPD software developed for spectroscopic data analysis [26]. The backgrounds were subtracted by using a Shirley background method; the deconvolution of the peaks was calculated by Voight functions, which are differential combinations of Lorentzian and Gaussian functions, as the Lorentzian functions represent the natural photoelectron process related broadening, Gaussian presents the machine and X-ray photon source related broadening. The separation of the W 4f doublet between the 5/2 and 7/2 peak was constrained to 2.15 and 2.18 eV for fully oxidized and vacancy concentrated W^5+^ oxidisation states, respectively. The doublet area ratio was constrained to 0.75 for 4f_5/2_/4f_7/2_. While the Lorentzian full width at half maximum (Lwod) drastically higher by being 0.3331 for W^5+^ in comparison to 0.15 for W^6+^, Gaussian full width at half maximum (Gwid) observed to steady state. This is due to a broad range of vacancies in the surface and disordered phase causing further broadening in the photoelectron peak [19]. According to McIntyre and Chan, the peak positions of the hydroxyls (OH^−^) and the oxide species (O^2−^) are usually 1.1–1.5 eV apart from each other, but the bonding of water molecules causes energy shifts of >3 eV. Similar values are also reported in the large volume of literature with binding energy values for different oxygen species [27,28]. However, none of those work reported chemisorbed species, although most of those studies were elucidated and conducted with utmost care. Limiting factors are the limitation of X-ray photon source and interest in the understanding on the chemisorbed species. This leads to the interpreted the chemisorbed species by researchers under the OH^−^/H_2_O groups only. In our work, it was possible to distinguish all four different oxygen species due to the 0.1/0.2 eV of the natural broadening of the X-ray sources, careful surface cleaning and low radiation damage and the high intensity of the synchrotron source. Based on the careful analysis the photoelectron peak properties for W 4f and O 1s were determined distinctly for OH^−^/H_2_O, chemisorbed species ($O_{2\left(ads\right)}^{-},\;O_{\left(ads\right),\;}^{-}O_{2\left(ads\right)}^{2-}$) and lattice oxygen ions in W^5+^ and W^6+^ oxidation states with corresponding ratios as well and provided in the Table 1, those values are going to be utilized in the succeeding sections.

#### 3.1.3. Non Destructive Depth Profiling of WO_3_ with Synchrotron Based Varying X-ray Photon Energies and Some Remarks on Oxidation State Analysis of WO_3_

**Figure 3:**
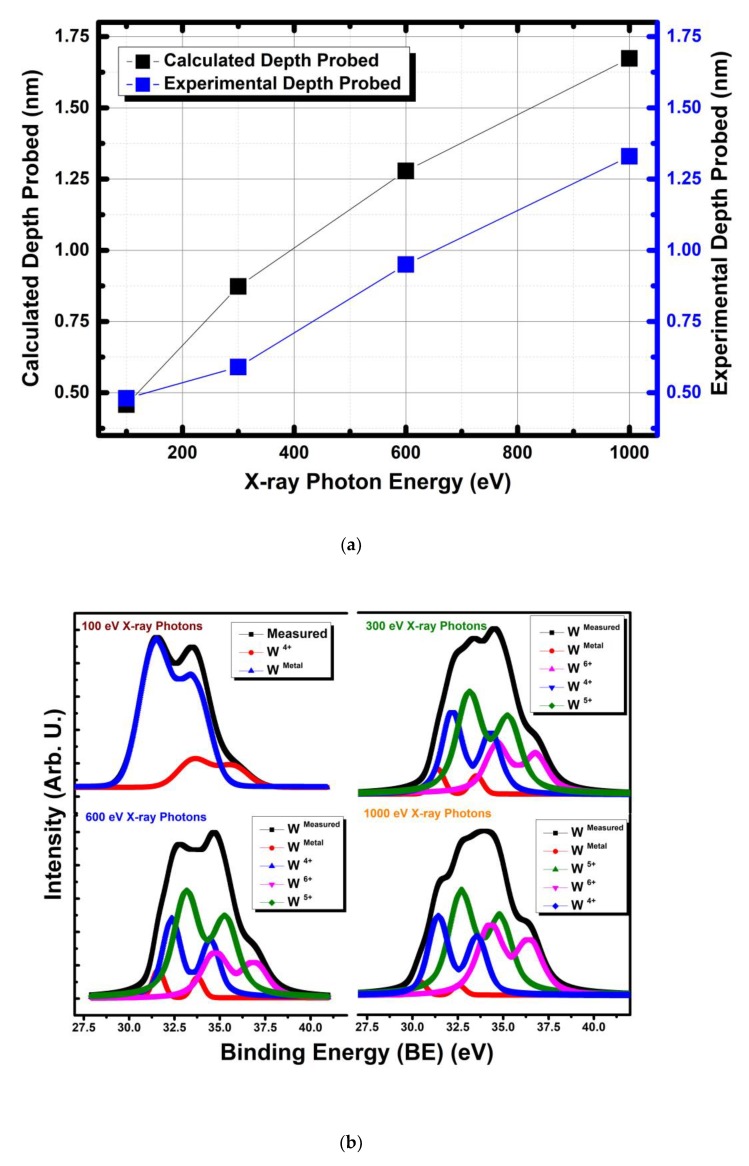

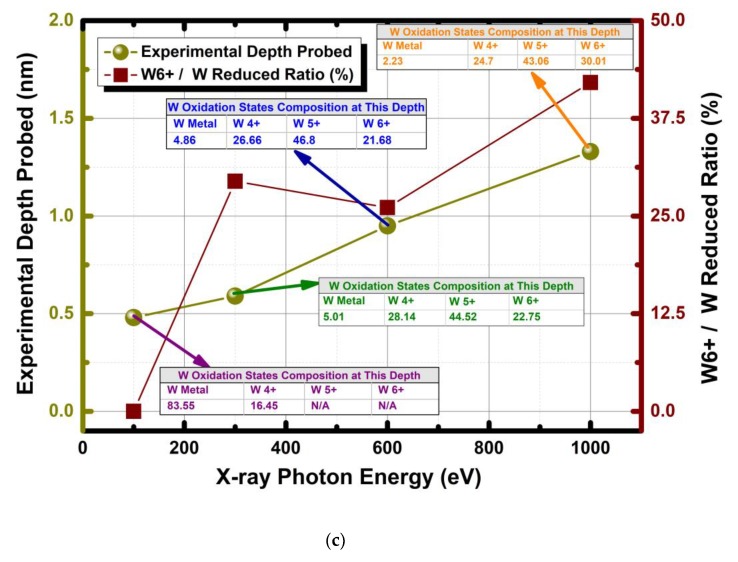
Non-destructive depth profiling of 600 °C substrate temperature deposited WO_3_ thin film with varying X-ray photon energy excitements. (**a**) The probed depth as a function of X-ray photon energy, (**b**) The deconvoluted W 4f photoelectron lines measured by 100, 300, 600 and 1000 eV X-ray photons. (**c**) Quantification of oxidation states as a function of probed depth with different X-ray excitations energies and change of the relative percentages of the fully oxidized state of tungsten (W^6+^) vs. the reduced states (W^5+^, W^4+^ and W^Metal^).

One of the main advantages of photoelectron emission (PES) experiments with synchrotron radiation is the tunable photon source that leads to very high surface sensitivity. Fine-tuning the X-ray photon energy will lead to the extraction of the electrons from the desired energy level with minimum escape depths, therefore it is possible to carefully and precisely examine the last few layers of the thin coatings and maximize the surface sensitivity thus increasing the focus on the immediate surface chemistry and chemisorbed oxygen species. Following the Low-dose experiments and the determination of the precise peak parameters, four different X-ray photon energies of 100, 300, 600 and 1000 eV were employed for non-destructive depth profiling of 600 °C deposited WO_3_ sample. Figure 3a shows the depth distance of the thin film from surface analyzed as a function of X-ray excitation photon energy. The depth distance values were derived in two different ways: (i) based on the ‘universal formula’ of the inelastic mean free path (IMFP) of electrons and (ii) experimentally determined values for W 4f electrons [7]. As could be noticed there is a discrepancy in the measured data for depth and the calculated value of the depth probed. The experimental IMFP values based analysis specific analysis of W 4f lead to the minimum depth probed 0.48 nm while the maximum 1.33 nm, as compared to the same minimum and maximum values of the calculated values are 0.45 and 1.67 nm based on IMFP formula. More importantly both values agree on the minimum depth probed in very close proximity of 0.45 nm, so practically it means surface adsorbed layers without carbon contamination and a very few tungsten planes were probed in the most surface sensitive configuration while in the least surface sensitive configuration it was probed as an average of ~1.33 nm, thus more and more WO_3_ crystal planes were involved in the photoelectron emission process. Considering the surface sensitive working principle of the chemical sensor based on semiconducting metal oxide (SMOs) architecture, 1.5 nm depth can be considered as a bulk for the sensor designer. It would be enlightening to compare those minimum depths achieved with laboratory based photon source systems. The laboratory based systems utilizes aluminum (Al) and/or magnesium (Mg) K_α_ radiations with X-ray energies of 1486.6 eV and 1253.6 eV, those energies which will lead to minimum depth probed 1.70 and 1.84 nm, respectively, and those values are significantly higher than 0.45 nm that could be achieved in the synchrotron based system. Both Al and/or Mg K_α_ radiations can provide mainly contributions from bulk where the chemical sensor reactions do not take place. Besides, limited resolution could be obtained from laboratory based systems. Basically, scientist should treat the data obtained from laboratory based system with more care and more theoretical involvement in order to provide remediation and comprehensive calculations and deconvolution analysis for reaching the alleviated precision.

Figure 3b shows the deconvolution analysis of the W 4f measured with four different X-ray photon energies. The result indicates that the existence of W^0^, W^4+^, W^5+^ and W^6+^ oxidation states. It is noteworthy to report that, the oxidation state of the WO_3_ thin films at the imminent surface is significantly different than the depth probed down to 1.33 nm. The alteration in the oxidation state of the tungsten was quantified with the precise parameters obtained from the preceding section for high precision deconvolution analysis. The amount of each oxidation states are given in mini tables as multiple insets in Figure 3c as a function of depth. The ‘dark yellow line’ in Figure 3c shows the composition of the thin film at each depth point measurement was taken. The imminent surface had no W^5+^ and W^6+^ phases. The composition was mainly W metallic phase and mixture of W^2+^ and W^4+^. This finding is different from most of the reported XPS data in chemical sensor literature found. In general, W^6+^ and fewer amounts including W^5+^ have commonly been reported as the chemical status of the surface. It could be seen in Figure 3c that each depth analyzed had different composition of tungsten oxidation states. The W oxidation state of W^6+^/W_Reduced_ is defined as the ratio between the amount of W^6+^ phase and the total reduced oxidation states of tungsten W^0^, W^4+^, W^5+^ shown in the Figure 3c as ‘wine’ in color. Figure 3c shows that as the probed depth increased the ratio of W^6+^/W_Reduced_ increases. As analysis depth increased to 0.6 nm, metallic state disappeared and W^4+^, W^5+^, W^6+^ oxidations states appeared to be dominating. The importance of that differentiating oxidations states in the chemical sensing reactions is significant, as they affect the sensor performance by creating active sites for adsorption for target gases as well as oxygen for chemisorption on the surface. This multiple oxidation state phenomenon might be the responsible for the case, n-type to p-type response switch behavior reported in the literature for and molybdate and tungstate based compositions under harsh environmental testing conditions leading to a reduction in the oxidation of the chemical sensor material [3,4,20,29].

### 3.2. Van Der Pauw (vdP) Electrical Resistivity Measurements and Chemical Sensor Testing for NO_2_

**Figure 4:**
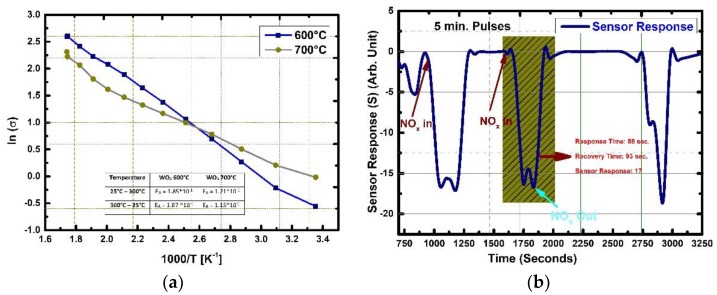
(**a**) Electrical conductivity as a function of the reciprocal of the temperature (1000/T) for CVD deposited WO_3_ thin films. The inset Table shows the activation energy values for WO_3_ thin films. (**b**) 600 °C deposited CVD WO_3_ thin film chemical sensor response curves as a function of gas concentration and time varying exposure times of 2.5 ppm of NO_2._

A brief electrical and chemical sensor testing was completed in order to correlate the results found in this work. It was seen that the surfaces chemistries of the films were different from the typical reported values for similar thin films. Figure 4a presents the natural logarithm of electrical conductivity (ln *σ*) as a function of the reciprocal of measurement temperature (1000/T). A distinctive linear part can be identified for both 600 and 700 °C deposited samples. This is due to the continuous contribution from extrinsic region due to oxygen vacancy ($V_{O}^{••})$ donor states onsets around 135 °C and accompanied by desorption of adsorbed species such as chemisorbed, water and hydroxyl groups that facilitated the smooth transition from extrinsic to the pseudo-intrinsic regions. It could be understood that surface vacancy concentration associated with the W^0^, W^2+^, W^4+^ and W^5+^ oxidations states. The WO_3_ thin films are n-type semiconducting oxides, due to the formation of donor $V_{O}^{••}$ donor states in the gap. The van der Pauw (vdP) electrical resistivity measurements are in good coherence with the LEEM and XPS-USP measurements. Donor states originate from oxygen vacancies and as noticed in LEEM images due to differentiating work function (Φ) measurements the dynamic interplay and reciprocating process of adsorption and desorption are limited to the surface region. The overall lower resistivity values measured for 700 °C deposited sample is directly related to the larger grain size, more dynamic surface for electron transfer and more carrier concentration of it. Figure 4a provides the activation energy values for 600 and 700 °C deposited WO_3_ thin films as an inset table. The increase in the deposition temperature from 600 °C to 700 °C, the activation energy decreased due to donor states in the gap consolidating their effects profoundly in the extrinsic region. For the 600 °C and 700 °C thin films, activation energies are 0.185 eV at the higher temperature site and 0.121 eV on the lower side, respectively. Furthermore, it is illustrated that the nonexistence of the transition between the extrinsic and the intrinsic conduction mechanisms without a distinct separation of the exhaustion region indicates that one dominant mechanism operates through the measurement. The WO_3_ thin film deposited at 600 °C was chosen to be tested for NO_2_ and sensing performance was provided in Figure 4b The gas testing experiments were conducted at 100 °C substrate temperature with different exposure times and concentrations of target gases to obtain a better understanding on the operational capabilities of the thin films for further detailed elaboration. The elementary reactions regarding the chemical sensing with an n-type of the semiconductor metal oxide are given in Equations (2)–(10) for an oxidizing gases with a special example of NO_x_ (NO_2_/NO). Adsorption of O_2_ either by physisorption and/or chemisorption, which consumes electrons as seen in Equations (2)–(5), subsequently oxidizing gas acts through and further extracts free electrons and/or replaces the chemisorbed species, oxidizing surface vacancies by donating O^−^ ions as seen in Equations (6)–(10). Nitrogen dioxide (NO_2_) is a strong oxidant and possesses electrophilic property leading to rapid adsorption on the metal-oxide surfaces. It can be seen that the oxidation of NO_2_ leads to the reduction of conduction electrons. 

(2) $$O_{2\left(gas\right)}+e^{-}\to O_{2\;\left(adsorbed\right)}^{-}$$

(3) $$O_{2\left(gas\right)}+2e^{-}\to 2O_{\left(adsorbed\right)}^{-}$$

(4) $$O_{2\left(adsorbed\right)}^{-}+e^{-}\to O_{2\left(adsorbed\right)}^{2-}$$

(5) $$O_{2\left(adsorbed\right)}^{2-}\to 2\;O_{\left(adsorbed\right)}^{-}$$

(6) $$NO_{2\left(gas\right)}+e_{\left(surface\right)}^{-}\to NO_{2\;\left(adsorbed\right)}^{-}$$

(7) $$NO_{2\left(gas\right)}+2e_{\left(surface\right)}^{-}+O_{2\left(adsorbed\right)}^{-}\to NO_{2\;\left(adsorbed\right)}^{-}+2\;O_{\left(adsorbed\right)}^{-}$$

(8) $$NO_{2\left(surface\right)}\to NO+O$$

(9) $$NO_{2\left(gas\right)}+V_{O}^{••}\to \left(V_{O}^{••}-O_{\left(adsorbed\right)}^{-}\right)+NO_{\left(gas\right)}$$

(10) $$NO_{2}+W^{5+}\overset{\left(adsorption\right)}{\to }\left(W^{6+}-NO_{2}^{-}\right)\overset{\left(desoprtion\right)}{\to }\left(W^{6+}-O^{-})+NO_{\left(gas\right)}\right)$$

The WO_3_ thin film sensor showed oxygen deficit n-type semiconducting metal oxide behavior against oxidizing gas, NO_2_, while the overall process was facilitated with surface defect centers as previously discussed especially W^4+^/W^5+^ points defects. The WO_3_ sensor showed an increase in resistance, and a decrease in the current once exposed to 2.5 ppm of NO_2_. The response and recovery times were short with a sensor response magnitude of 17, while the sensor showed successful sensing with multiple exposures to target gas. The maximum sensor response was 19 towards 2.5 ppm of NO_2_. NO_2_ has the capability to react with the metal-oxide surface with the presence of O_2_ and/or absence of it as given in the Equations (6), (7) and (10). NO_2_ molecule can be absorbed on fully oxidized sites (Equation (6)), however; the dissociation of it happens at the oxygen vacancy sites (Equations (9) and (10)). As can be followed from the Kroger-Vink defect equations, W^4+^/W^5+^ defect centers together with oxygen vacancy sites ($V_{O}^{••}$) helps adsorption and dissociation of NO_2_ to O and NO (Equations (8)–(10)), so promotes the oxidation of the surface at comparatively low temperature 100 °C.

### 3.3. Near Ambient Pressure (NAP) XPS Analysis of SnO_2_ and Quantification of Chemisorbed Oxygen Species as a Function of O_2_ and CO Exposure and Temperature

**Figure 5:**
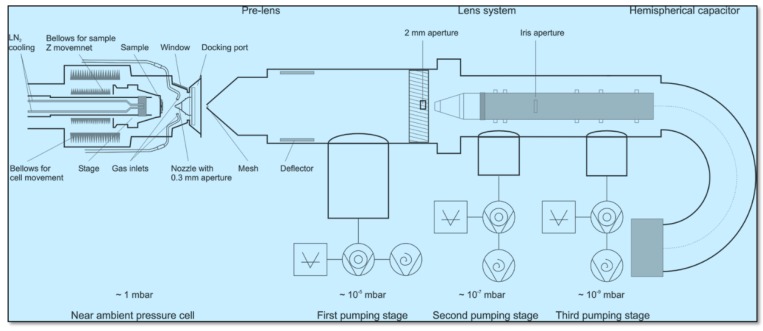
A schematic of the NAP-XPS system utilized for high temperature up to 400 °C and high pressure CO and O_2_ exposures and simultaneous X-ray photoelectron spectroscopy (XPS) data collection.

NAP-XPS is designed for the chemical analysis of surfaces in the presence of reactive working atmosphere in the mbar range up to 10 mbar via X-ray photoelectron spectroscopy (XPS). In-situ monitoring of surface reactions, dynamic processes, chemical reactions, structural dynamics, and materials with a high vapor pressure are among the areas that can be studied. Nevertheless, until now experimental studies focusing on surface chemistry by employing photoelectron spectroscopy have been restricted to the before and after gas exposure, not during the exposure itself. The reason for this limitation is mainly due to the typical interest in the gas pressure which lies in the mbar range, which is not suitable for conventional XPS instrumentation that requires ultra-high vacuum (UHV) conditions. Figure 5 presents the schematic of the NAP-XPS system utilized in the current work. The experiment was performed using the laboratory NAP-XPS system (SPECS Surface Nano Analysis, GmbH Germany) in Prague, Czech Republic. The setup consisted of a main UHV Analysis chamber that was equipped with a monochromatic aluminum (Al) K_α_ X-ray source of high intensity, a multichannel electron energy analyzer (Specs Phoibos 150) coupled with a differentially pumped electrostatic pre-lens system. The base pressure in the analysis chamber was 1 × 10^−9^ mbar. In the NAP-XPS setup it is possible to monitor the chemical interaction between a solid surface and reactive gases. NAP-XPS will lead to an understanding of the effect of the oxygen partial pressure on the oxidation state, stoichiometry, work-function (Φ), valence band, chemisorbed oxygen ion status and concentration and defect analysis.

A key question significantly relevant for the chemical sensor operations is which part of the oxygen sublattice on the surface interacts with the target gas species (SO_2_, H_2_S, CO, H_2_, NO_x_, etc.) and/or in which turn the interaction takes place. In the current work, NAP-XPS was utilized for chemical sensor analysis and CO was used as a target gas for 20 nm thick SnO_2_ film in order to understand the reducing gas and oxygen network interaction on the surface. Experiments were conducted under operational conditions with realistic partial pressures of CO and O_2_ ranging from 1 mbar to 5 mbar at realistic chemical sensor operation temperatures ranging from room temperature (25 °C) up to 400 °C. SnO_2_ characterization with NAP-XPS will also provide understanding over the degradation mechanism as a function of temperature and reducing gas partial pressure by inducing oxygen vacancies, and/or humidity formation, reduced states and its effect on the stoichiometry. This type of analysis has not yet been reported for chemical sensor applications in the open literature [16].

**Figure 6:**
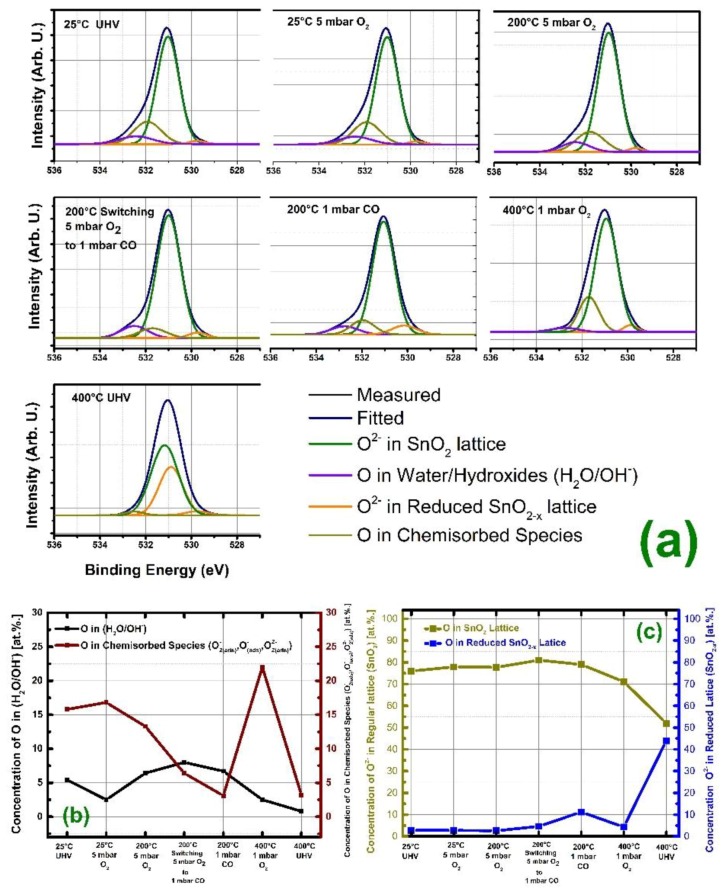
(**a**) Quantification of different oxygen species upon exposure to various amounts of O_2_ and CO gases at different temperatures completed on deconvolution analysis with precise parameters obtained through Lowdose PM4 measurements in the preceding section. (**b**) shows the concentration of O^2−^ ions found in water/hydroxyl groups and chemisorbed species while (**c**) presents the atomic concentration of the O^2−^ ions detected in the regular lattice (SnO_2_) positions and the reduced lattice positions (SnO_2-X_) as a function of temperature, Oxygen and CO gas concentrations. The detailed information regarding the binding energy and concentration can be found in Table 2.

It is the first that time in-situ analysis of the SnO_2_ under realistic CO partial pressure to observe changes in the oxygen and tin (Sn) sublattice on the chemical sensor surface under 1 mbar of CO exposure from room temperature up to 400 °C in 30 min of intervals of gas exposure cycles was realized. Figure 6a gives the oxygen (O) 1s spectrum taken under six different experimental conditions for SnO_2_ thin film, while Figure 6b details the amount of chemisorbed oxygen species and OH^−^/H_2_O groups as a function of temperature, O_2_ and CO partial pressures. Those conditions are explicitly given in Table 2, which also provides the concentration and photoelectron binding energy (BE) values for the different oxygen species. Figure 6b,c depict quantification of the concentrations of the lattice oxygen (in SnO_2_), reduced lattice oxygen (in SnO_2−x_), chemisorbed species ($O_{2\left(ads\right)}^{-},\;O_{\left(ads\right),\;}^{-}O_{2\left(ads\right)}^{2-}$), and OH^−^/H_2_O functional groups in atomic percentage scale (at.%.). The increase in chemisorbed ion concentration was detectable as the samples were exposed to 5 mbar O_2_ at 25 °C for 30 min. The treatment with 5 mbar O_2_ at 200 °C led to slight decrease in chemisorbed oxygen ions accompanied by an increase in the OH^−^/H_2_O related surface species, it could be argued that, this represents the repositioning of the surface and the screening effect of the upward diffusing and/or partial termination of most outer layers of OH^−^/H_2_O groups and inward movement of the single and double charged chemisorbed species, the removal of screening by OH^−^/H_2_O groups leading the detection of the deeper layers of lattice, and more adsorbed layers of the chemisorbed species.

Exposing the SnO_2_ thin film to 1 mbar of CO at 200 °C, drastically decreased the amount of chemisorbed species down to 5 at.% within 2 min of exposure time, while no reduction in the regular lattice site was observed in addition to that, no increase in the reduced lattice related oxygen was detected in the meantime, however sustained exposure to 1 mbar CO at 200 °C for 30 min showed its detrimental effect and reduction in SnO_2_ was clearly detected after 30 min exposure of CO. Figure 6c presents the Sn^4+^ and Sn^2+^ concentrations as function of temperature, O_2_ and CO pressures. The amount of oxygen ions connected to the Sn^2+^ sublattice was increased to 11.2 at.%. After this step, sample was exposed to 5 mbar of O_2_ at 400 °C for 30 min., and a high level of recovery in the Sn^4+^ oxidation state was observed. The concentration of chemisorbed species increased to 23 at.%., while the reduced Sn^2+^ related phase was again less than 3 at.%, however terminating the 5 mbar of O_2_ at 400 °C showed destructive effect on the samples stoichiometry and the Sn^2+^ related phase increased to 44 at.%. The severe reduction caused a detection of the intermediate oxidation states between Sn^2+^ and Sn^4+^ with high intensity, both chemical states are as shown as ‘yellow’ in colour in Figure 6a under 400 °C UHV measurements graph for the O 1s position. This was accompanied by the sudden decrease in the chemisorbed species as well and the total termination of water and hydroxyl groups.

**Figure 7:**
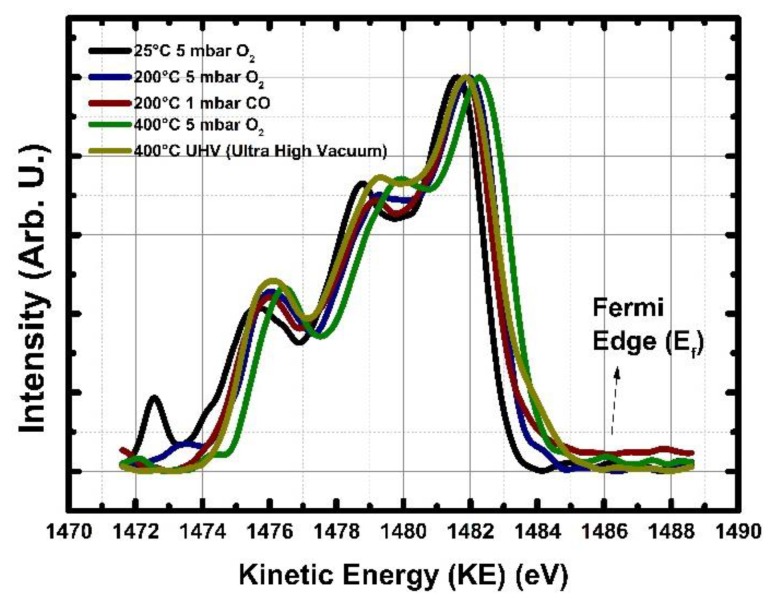
Valance band spectrum of the SnO_2_ thin film as a function of O_2_ and CO exposures and temperature.

Modification of the electronic structure can be clearly observed by the utilization of spectroscopic techniques, for example, in the valance band photoemission spectrum presented in Figure 7. The valance band spectrum verified that, 5 mbar O_2_ at 200 °C has an effect of an ion sputtering like and increasing the electron population by decreasing OH^−^/H_2_O groups, a similar effect in valance band was also observed after exposing to CO at 200 °C, however this time a steep decrease in the chemisorbed species in the concentration was observed. The CO exposure at 200 °C affected the chemical state of the compound as well, ensued reduction in oxidation state of the SnO_2_ occurred at 400 °C. 5 mbar O_2_ partial pressure treatment for 30 min mainly oxidized the reduced states, however complete recovery could not be reached. More occupancy on the Sn 5s conductance band was detected in the valance band very end of the spectrum very close to the Fermi level, on the high kinetic energy side. The kinetic energy scale in Figure 7 is referenced to the Fermi level (EF), which is located close to the lower edge of the conduction band in this n-type semiconductor. The valence band maximum is located at approximately ~3.1 eV, consistent with the band gap of SnO_2_. As seen in the Figure 7, mainly Sn 5s, Sn 5d and O 2p states contribute to the valence band of SnO_2_ while the conduction band contains mainly 5s states. CO exposure gives rise to the defect state 3.4 eV referenced to the Fermi level. Thus, the reduction of the surface results mainly in the occupation of the empty Sn 5s states in Sn (IV). 

**Figure 8:**
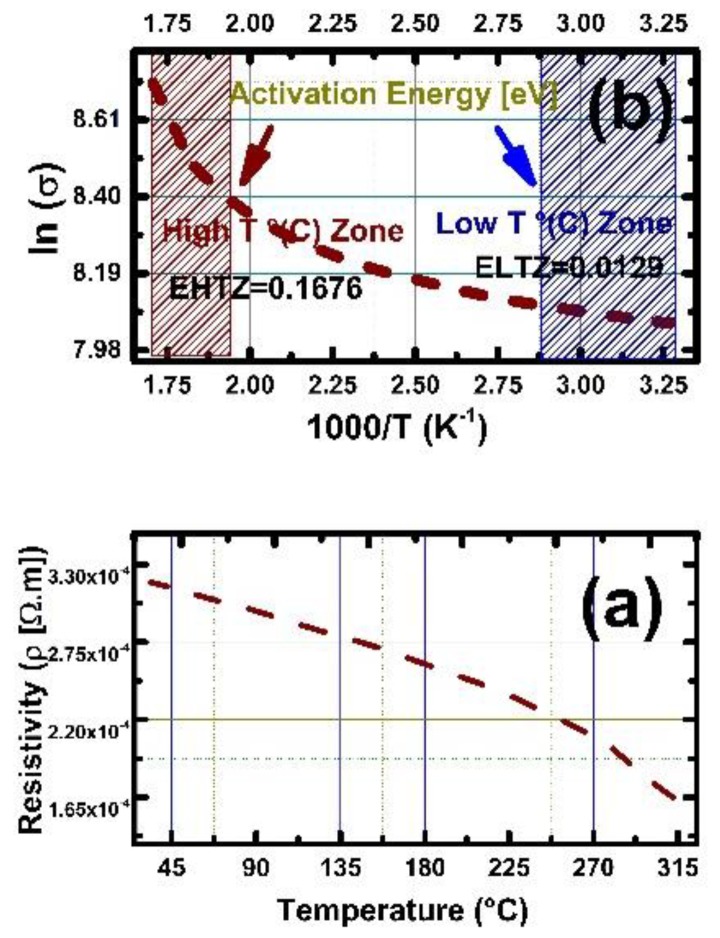
(**a**) Dynamic resistivity of 20 nm thick PEALD SnO_2_ as a function of temperature under atmospheric conditions. (**b**) Electrical conductivity as a function of reciprocal of temperature (1/T) for PEALD deposited SnO_2_ thin films.

Figure 8a shows the resistivity values of SnO_2_ thin films. The thin film thickness is again 20 nm and the dynamic resistivity as a function of temperature were registered under the environmental atmospheric conditions. The experiments were completed 25–300 °C temperature window. As the Figure 8a represents, an increase in temperature, continuously decreased the resistivity values for SnO_2_ thin film. The measured electrical resistivity value at room temperature is 3.16 × 10^−4^ (Ω.m). The resistivity declined to 2.62 × 10^−4^ (Ω.m) at 150 °C. While the tempo of the decrease in resistivity diminished a further increase in the temperature brought a decrease in the resistivity, at 300 °C the resistivity value is 1.76 × 10^−4^ (Ω.m). After 150 °C testing temperature, the rhythm of decline was minor due to annealing of some of oxygen vacancies *(*$V_{O}^{\cdot \cdot }$*)* on the surface, NAP-XPS analysis also indicated that starting from 200 °C, the oxygen lattice replenishment rate is high in chemisorbed and surface adsorbed species as well. The electrical resistivity values stated for thin SnO_2_ films deposited by different techniques at room temperature are as follows for EB-PVD, ALD, Sputtering, spray pyrolysis, CVD and PVD; 10^−4^, 2 × 10^−4^, 2.5 × 10^−5^, 2 × 10^−5^, 1 × 10^−3^, 5 × 10^−5^, 5 × 10^−4^, 4.5 × 10^−4^ (Ω.m), respectively [30,31,32,33,34]. The considerable deviations among the reported resistivity values in the open literature is linked to different deposition techniques and/or post-deposition processing leading different microstructural formations, surface chemistry properties and more importantly different surface varying stoichiometries [31,34,35,36].

SnO_2_ thin film showed reversible electrical resistance values after three successive experimental runs. In other words, hysteresis formation was not observed due to a temperature or chemical driven process, apart from reversible adsorption and desorption of O^2−^ species. Hysteresis free, resistivity performance of all thin films further facilitates their usability for chemical sensor applications. The results included in this report indicate that, SnO_2_ thin film is an n-type semiconducting oxide, due to formation of donor O^2−^ donor states in the gap [30,33]. Reversible surface point defect dynamics quantified by XPS and under operation conditions by NAP-XPS pave the ground for stabile resistivities experienced after 200 °C. Figure 8b presents the natural logarithm of electrical conductivity (ln *σ*) as a function of reciprocal of measurement temperature (1000/T). Two distinctive linear parts can be identified as positioning themselves in the high and low temperature regions, and those can be associated with different electronic conduction pathways. The transition between extrinsic and intrinsic electrical conduction mechanisms merged in the middle temperature region without distinctive separation identifying the exhaustion region. This is due to the continuous contribution from extrinsic region due to oxygen vacancy *(*$V_{O}^{\cdot \cdot }$*)* donor states, associated with Sn^2+^ states, onsets around 135 °C and accompanied by desorption of adsorbed species such as chemisorbed, water and hydroxyl groups that facilitated the smooth transition from extrinsic to the pseudo-intrinsic region. In Figure 7, it is seen that continuous shoulder formation on the higher kinetic energy side associated with sustained development of Sn^2+^ states and reoxidation back to Sn^4+^ state.

## 4. Conclusions

WO_3_ and SnO_2_ were analyzed by means of advanced synchrotron based XPS, UPS, NAP-XPS techniques under realistic working conditions for semiconducting metal oxide (SMO) chemical sensors. The chemical nature and oxidation state of the WO_3_ and SnO_2_ thin films were explored at different measurement depths from imminent surface to down to 1.5 nm depth from the surface. It was observed that, at the most surface sensitive mode, the composition of the WO_3_ is mainly metallic (W^Metal^), however increasing data accusation depth to 1 nm and beyond drastically changed the chemical status of the WO_3_ film and mainly the W^4+^, W^5+^ and W^6+^ oxidation states were detected and tabulated as a function of depth probed. The amount of OH^−^/H_2_O groups, chemisorbed oxygen species (**(**$O_{2\left(ads\right)}^{-},\;O_{\left(ads\right),\;}^{-}O_{2\left(ads\right)}^{2-}$**)**) and lattice oxygen ions (O^2−^) were quantified and graphed/tabulated at 25 °C, 200 °C and 400 °C under 1 mbar CO and 5 mbar O_2_ exposures for SnO_2_ thin films for the first time under realistic conditions. It was possible distinguish all four different oxygen species due to 0.1/0.2 eV of natural broadening of the X-ray sources, careful surface cleaning and low radiation damage and high intensity of specifically chosen synchrotron source. Those measurement temperatures and partial pressure of O_2_ and CO are realistic working conditions for the chemical gas sensor testing. The electrical resistance and sensor testing experiments were also completed in order to connect the surface chemistry analysis with gas sensing behavior. A comparison and evaluation of different spectroscopic/microscopic characterization techniques for analysis of chemical sensors points out that, in the near future NAP-XPS and synchrotron based techniques will contribute more and more to basic understanding of the chemical sensing mechanism of complex semiconducting metal oxides (SMOs) within integral design for addressing future selectivity/sensitivity demands for the sensor designers. 

## Figures and Tables

**Table 1:** Low dose synchrotron analysis of O and W positions from WO_3_ thin film.

	W^6+^ 4f_7/2_	W^5+^ 4f_7/2_	O in Water/Hydroxide(H_2_O/OH^−^)	O in Chemisorbed ($O_{2\left(ads\right)}^{-},\;O_{\left(ads\right),\;}^{-}O_{2\left(ads\right)}^{2-}$)	O Lattice (in WO_3_)	O Lattice Reduced (in WO_3−x_)
Binding Energy [eV]	35.66	34.44	533.49	532.37	531.41	530.31
Doublet Separation [eV]	2.15	2.17	NA	NA	NA	NA
Full Width Half Maximum Values (FWHM) [eV]	0.81 (gwid) 0.15 (lwid)	0.77 (gwid) 0.31 (lwid)	1.96	1.60	1.21	0.8
Relative amount [at.%.]	93.88	6.12	1.52	29.73	40.58	28.17
Natural broadening of X-ray photons [eV]	0.06	0.06	0.1	0.1	0.1	0.1

**Table 2:** Concentration and binding energies of different oxygen species upon exposure to 5 mbar of O_2_ and 1 mbar of CO at 25 °C, 200 °C and 400 °C.

O 1s Spectrum upon Exposure to Below Given, O_2_, CO Gases at Different Temperatures	O in Water Hydroxides (H_2_O/OH^−^) BE (eV) and Concentration [at.%.]	O in Chemisorbed $\left(O_{2\left(ads\right)}^{-},\;O_{\left(ads\right),\;}^{-}O_{2\left(ads\right)}^{2-}\right)$ BE (eV) and Concentration [at.%.]	O lattice (in SnO_2_), BE (eV) and Concentration [at.%.]	O lattice Reduced (in SnO_2_), BE (eV) and Concentration [at.%.]
25 °C UHV (Ultra High Vacuum)	532.4/5.4	531.9/15.8	531.0/76	530.4/2.8
25 °C 5 mbar O_2_	532.8/2.5	532.0/16.8	530.9/77.9	529.5/2.8
200 °C 5 mbar O_2_	532.4/6.4	531.8/13.3	530.9/77.7	529.8/2.6
200 °C 5 mbar O_2_ to 1 mbar CO	532.5/8.0	531.7/6.4	530.9/81	529.7/4.6
200 °CK 1 mbar CO	532.7/6.7	531.9/3.1	531.0/79	530.1/11.2
400 °C 5 mbar O_2_	532.7/2.5	531.7/22.1	530.9/71.1	529.8/4.3
400 °C UHV (Ultra High Vacuum)	NA/NA	532.5/3.2	531.1/52.3	530.9 and 529.7/44.5 (including both Sn^2+^ and Sn^X^, 2 < x < 3 phases both shown to be yellow in the Figure 6a)
